# Peroxisome Proliferator-Activated Receptor Delta Agonist (PPAR- δ) and Selective Androgen Receptor Modulator (SARM) Abuse: Clinical, Analytical and Biological Data in a Case Involving a Poisonous Combination of GW1516 (Cardarine) and MK2866 (Ostarine)

**DOI:** 10.3390/toxics9100251

**Published:** 2021-10-07

**Authors:** Pascal Kintz, Laurie Gheddar, Camille Paradis, Mickael Chinellato, Alice Ameline, Jean-Sébastien Raul, Magali Oliva-Labadie

**Affiliations:** 1 Institut de Médecine Légale, 11 rue Humann, 67000 Strasbourg, France; l.gheddar@gmail.com (L.G.); ameline.alice@gmail.com (A.A.); js.raul@unistra.fr (J.-S.R.); 2 Centre Antipoison et de Toxicovigilance, Groupe Hospitalier Pellerin, Place Amélie Raba Léon, CEDEX, 33076 Bordeaux, France; camille.paradis@chu-bordeaux.fr (C.P.); magali.labadie@chu-bordeaux.fr (M.O.-L.); 3 Service des Urgences, Centre Hospitalier de Marmande, 76 rue du Dr Courret, 47200 Marmande, France; n.chinellato@gmail.com

**Keywords:** GW1516, cardarine, MK2866, ostarine, SARM, PPAR- δ, clinical, blood, hair, doping

## Abstract

A 43-year-old male, sport coach, presented him-self at the Emergency unit of a local hospital for epigastric pain, myalgia pain and severe headache. He claimed having used for some days a combination of GW1516 (cardarine), a peroxisome proliferator-activated receptor delta agonist (PPAR- δ) and MK2866 (ostarine), a selective androgen receptor modulator (SARM) to gain skeletal muscles. Cytolysis with marked increase of alanine aminotransferase or ALT (up to 922 UI/L) and aspartate aminotransferase or AST (up to 2558 UI/L) and massive rhabdomyolysis with elevated creatine phosphokinase or CPK (up to 86435 UI/L) were the main unusual biochemistry parameters. Using a specific liquid chromatography coupled to tandem mass spectrometry method, cardarine and ostarine tested positive in blood at 403 and 1 ng/mL, respectively. In urine, due to extensive metabolism, the parent GW1516 was not identified, while ostarine was at 88 ng/mL. Finally, both drugs were identified in hair (2 cm in length, brown in colour), at 146 and 1105 pg/mg for cardarine and ostarine, respectively. This clearly demonstrates repetitive abuse over the last 2 months. Asthenia was persistent for 2 weeks and 6 weeks after the admission, the subject fully recovered.

## 1. Introduction

The abuse of performance-enhancing drugs is not a recent phenomenon. Since the 60’s, anabolic steroid drugs, such as testosterone derivatives, have been used to promote muscle growth. Nandrolone, trenbolone, stanozolol or boldenone and more recently tetrahydrogestrinone are associated with famous doping cases, including state doping programs. These products were manufactured by pharmaceutical groups and were available, as oral tablet forms but mostly as oily injectable preparations. In most cases, they were available from pharmacies in some legal less-regarding countries. With the development of e-shops on the Internet and the continuous research for testosterone substitutes, new pharmacological classes have emerged, including selective androgen receptor modulators (SARMs) such as ligandrol (LGD-4033), testolone (RAD140), ostarine (MK2866) or andarine (S-4) and peroxisome proliferator-activated receptor delta agonists (PPAR- δ) such as cardarine (GW1516 or GW501516) [1,2,3]. 

These drugs are abused because athletes and amateurs have claimed that they increase lean body mass, increase strength, increase aggressiveness and lead to a shorter recovery time between workouts. SARMs present high anabolic potency, in addition to limited androgenic effects [4,5]. PPAR- δ are lipid-activated transcription factors playing important regulatory functions in development and metabolism. Activation of the receptor promotes fatty acid burning by up-regulation of fatty acid uptake, ß-oxidation and energy uncoupling [6,7]. According to the World Anti-Doping Agency (WADA), SARMs and PPAR- δ are prohibited at-all times (in- and out-competition) as they are listed on the prohibited list under sections S1.2 (other anabolic agents) and S4.5 (metabolic modulators), respectively [8]. The number of adverse analytical findings involving SARMs and PPAR- δ during doping controls is continuously increasing in the recent years [9] and many cases of dietary supplements contamination have been reported [10,11].

SARMs have been proposed to treat hypogonadism, muscular atrophy or osteoporosis but the different compounds did not demonstrate enough safety and efficacy to gain clinical approval in the United States or in Europe. Indeed, they can produce heart attack, liver damage and blood clots [12,13]. Due to the induction of intestinal adenoma in rats during the initial clinical trials, Glaxo Smith Kline decided to stop all investigations involving GW1516 [14].

**Figure 1:**
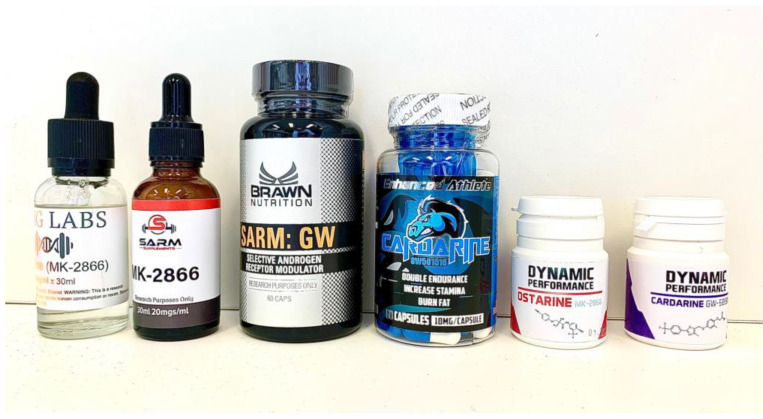
Typical material sold on the Internet.

Ostarine and cardarine are mostly available as liquid solutions and tablets (Figure 1), but it is possible to buy larger quantities as bulk material (powders), essentially in China. 

Even though SARMs and PPAR- δ are easily available on the Internet, very few poisoning cases have been reported in the medical literature. Several reasons can account for this under-representation, including the absence of recreational effects but this situation is mainly due to the poor interest of analytical laboratories to test for SARMs and PPAR- δ. Indeed, anti-doping accredited laboratories have published quite most detection methods in urine [15,16,17] and they do not perform tests for clinical samples. As a consequence, in the limited case reports involving SARMs or PPAR- δ, there is no toxicological data (identification of the substance and evaluation of its concentration). Testing for ostarine [9,18,19,20] and GW1516 [21] in human hair is the privilege of our laboratory as no other citation is available in the scientific literature.

This technology was used to document a poisoning case after repetitive consumption of ostarine and GW1516, requiring the hospitalization of the abuser. 

## 2. Case Report

A 43-year-old male, sport coach, presented him-self at the Emergency unit of a local hospital for epigastric pain, myalgia pain, severe headache and brown urine. He claimed having used for some days a combination of GW1516 (4 days, 20 mg per day) and MK2866 (1 day, 20 mg) to gain muscle mass. This occurred 10 days before he went to the hospital. Both products were bought on the Internet (10 g of GW1516 and 46 g of MK2866 for a total of 200 euros). The day before he went to the hospital, he cycled 120 km. Vital signs included a blood pressure of 135/70 mmHg and a heart rate of 76 beats per minute and no respiratory distress (SpO_2_ at 99%). The ECG was normal. The abdomen was supple but painful. Initial laboratory tests showed serum creatinine of 109 µmol/L (clearance at 76 mL/min), elevated liver enzymes (alanine aminotransferase or ALT 966 UI/L, aspartate aminotransferase or AST 1000 UI/L), normal bilirubin and creatine phosphokinase or CPK elevated at 10,000 UI/L. Blood (green Vacutainer), urine without preservative and hair (2 cm in length, brown in colour) were immediately collected and sent to the laboratory for toxicological investigations. Some hours later, rhabdomyolysis worsened with a rise of CPK at 57,000 UI/L. Treatment consisted of intra-venous (IV) re-hydrating at 3 L per 24 hours. The next day, biochemical tests included CPK at 86435 UI/L, ALT at 922 UI/L and AST at 2558 UI/L. No kidney insufficiency was noticed and urine started to become clearer. Muscle pain remained but abdominal pain was gone. The subject was allowed to turn home, despite the installation of an asthenia that lasted for 15 days. Five days later, during a control, CPK were at 1765 UI/L, ALT at 726 UI/L and AST at 331 UI/L. Normal values were obtained after 2 weeks. Six weeks after the event, the subject totally recovered. Both white powders used by the subject were also submitted to the laboratory for confirmation of identity and determination of purity.

## 3. Materials and Methods

Powders were diluted in methanol to obtain a 10 mg/mL solution. This solution was compared to reference standards (supplied as powders) obtained from Cayman Chemical Company (Ann Arbor, MI, USA). 

The biological specimens (blood, urine and hair) were tested by liquid chromatography coupled to tandem mass spectrometry (LC-MS/MS), using previously described methods for ostarine [18,19] and cardarine [20]. Briefly, 1 mL of blood or hydrolyzed urine (with 20,000 UI of ß-glucuronidase at pH 5.2) was extracted in 1 mL borate pH 9.5 buffer and 5 mL of diethyl ether/dichloromethane/hexane (50/30/20, *v*/*v*) in presence of 100 ng of bicalutamide-d_4_ used as internal standard. After centrifugation, collection of the supernatant and its evaporation to dryness, the residue was reconstituted in 30 µL of 5 mM ammonium formate buffer adjusted at pH 3. 

The whole hair strand was first decontaminated by 2 baths of 5 mL dichloromethane for 2 min and then cut into small pieces (< 1 mm) with small scissors. Drugs were extracted from 20 mg decontaminated cut hair in the presence of 1 ng of bicalutamide-d_4_, after overnight incubation in 1 mL of pH 9.5 borate buffer at 40 °C. After cooling, the mixture was extracted as blood. 

The conditions of use of the equipment (Waters Acquity class I ultra-high performance liquid chromatography and XEVO TQS micro triple quadrupole mass spectrometer) have been described in previous papers [18,19,20]. Briefly, separation was achieved using a Waters Acquity HSS C18 column (150 × 2.1 mm × 1.8 µm) at 50 °C using a gradient elution with pH 3 formate buffer (phase A) and 0.1% formic acid in acetonitrile (phase B) and a flow rate at 0.4 mL/min. The initial gradient was 60% phase A for 30 s to 5% at 1.5 min, kept for 1.5 min and a return at 4 min, maintained for 2 min. The injection volume was 3 µL. Ionization was achieved using electrospray in the negative ionization mode (ES-). Collision energy and cone voltage were adjusted to optimize the signal for the 2 most abundant product ions and are presented in Table 1.

## 4. Results

The analysis of the powders confirmed the identity of the drug. No other organic chemical was identified in each item. Ostarine purity was 28%. Cardarine purity was 100%. Purity was established by comparing the chromatographic response (area) obtained after injection of a powder solution at 10 mg/L in methanol with a solution of certified standard at the same concentration.

The major validation parameters of the analytical method in blood and hair are presented in Table 2. The validation was achieved using the ISO 17025 guidelines.

To date, and to the best of the authors’ knowledge, ostarine or cardarine detection has not been reported in clinical specimens. The biological samples were submitted to LC-MS/MS and both drugs tested positive in blood and hair, while only ostarine was identified in urine. This is not surprising as it has been reported that cardarine is extensively metabolized and that the parent drug is measurable only for a very short period of time, i.e., no more than 5 days [22,23]. For example, Sobolevsky et al. [22] identified the parent drug for 3 days after oral administration of 15 mg to a volunteer. Given the urine specimen of the present subject was collected 10 days after drug discontinuation, it seems consistent that the parent compound was no more present. Major metabolites include the oxidized GW1516-sulfoxide and the GW1516-sulfone, the later being found in notably higher internal standard than the first one during controlled studies [22]. Unfortunately, these metabolites are not available from reference material suppliers. To date, only WADA accredited laboratories have published data involving these by-products. Concentrations obtained in the submitted biological specimens are reported in Table 3.

**Figure 2:**
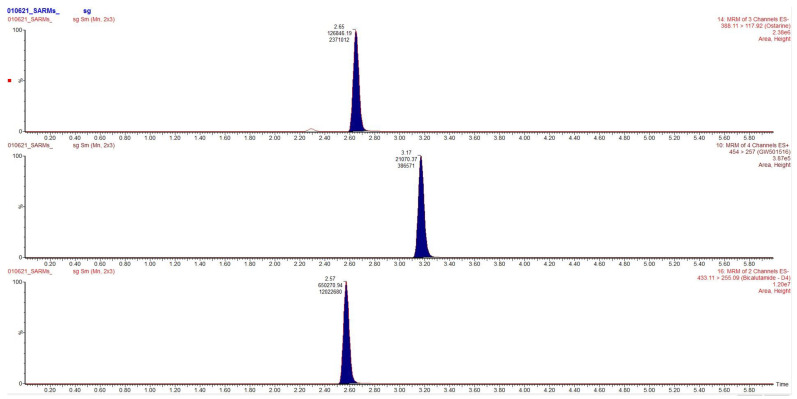
Chromatogram obtained after extraction of the blood. From top to bottom: ostarine (1 ng/mL), cardarine (403 ng/mL) and the internal standard.

**Figure 3:**
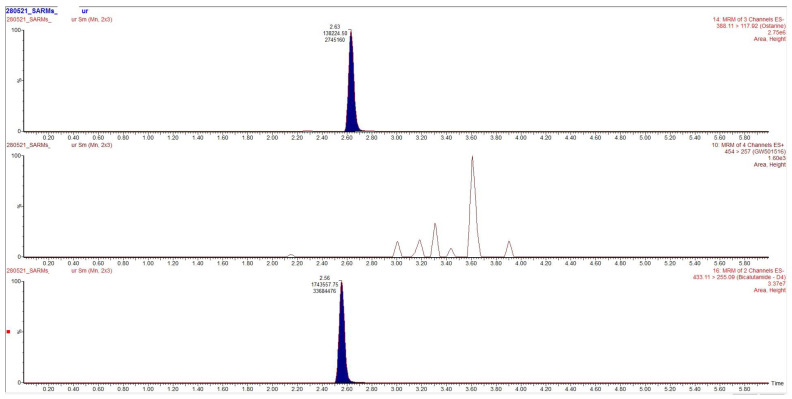
Chromatogram obtained after extraction of the urine. From top to bottom: ostarine (88 ng/mL), cardarine (not detected) and the internal standard.

**Figure 4:**
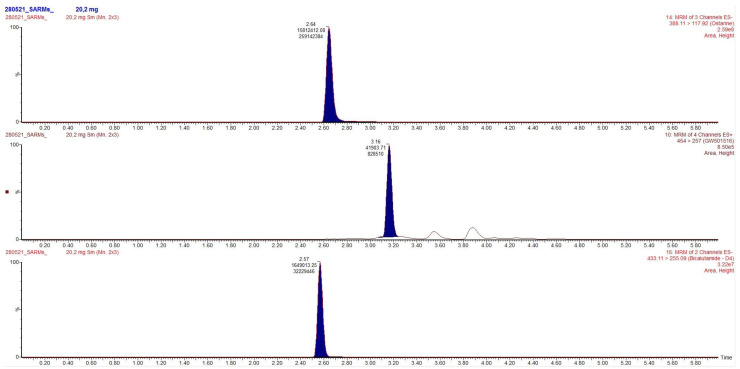
Chromatogram obtained after extraction of the hair. From top to bottom: ostarine (1105 pg/mg), cardarine (146 pg/mg) and the internal standard.

Chromatograms obtained after extraction of blood, urine and hair are presented in Figure 2, Figure 3 and Figure 4, clearly demonstrating suitable sensitivity of the method.

## 5. Discussion

The subject was admitted to the hospital for severe pain. Major biochemical disruption included rhabdomyolysis and liver cytolysis, as evidenced by enhanced creatine phosphokinases and transaminases. The use of SARMs and PPAR- δ and subsequent rhabdomyolysis has not yet been described. However, it is common to observe rhabdomyolysis in bodybuilders, abusing long-term anabolic steroids, a pharmacological class that is close to SARMs [24,25]. Repetitive abuse of clenbuterol, a ß2-agonist with anabolic properties can also induce rhabdomyolysis [26]. It must be emphasized that rhabdomyolysis is commonly associated with parallel rises in aminotransferases, as these enzymes are also present in muscle.

Even though nothing has been reported for cardarine, human liver injury has been described with ostarine [12,27,28]. However, in these cases, ostarine was always abused on a long-term basis, for several weeks. This is in total contradiction with the claims of the subject, reporting intake of ostarine for 1 day and intake of cardarine for 4 days. The discrimination between these 2 opposite situations was achieved with the hair test results. 

Indeed, although hair is not a routine specimen for the WADA, its use as a specimen of investigation is accepted in clinical toxicology [29] and in forensic toxicology [30]. Hair testing is a useful measure of drug intake of an individual, in any situation in which a history of past rather than recent drug use is expected, as it reflects consumption over a long period of time. For practical purposes, it is commonly accepted that each cm of head hair represents the growth, and therefore drug accumulation, for one month. Hair test results allow establishing a retrospective calendar of drug exposure over weeks to months based on a standard head hair growth rate of 1 cm per month. Using segmental hair analysis, statements about the course of drug intake and chronological correlations are possible. While a constant regular profile along the hair shaft is in favour of permanent drug use, any variation of concentration indicates a change in drug intake. Given the submitted material from the subject was limited, there was no attempt to perform segmental analysis in this case. The advantages of hair tests for active substances over blood and/or urine testing include non-invasive and ease of collection to prevent adulteration or substitution.

It must be acknowledged that a single exposure to SARMs is not detectable in hair [9], except for GSK2881078 [31]. There is no data about GW1516 detection after a single exposure. In a case of a subject abusing cardarine for improving athletic performances, concentrations of 32 and 22 pg/mg were measured in 2 × 2 cm segments [21]. Ostarine has been identified on several occasions, including doping challenges or drug trafficking [9,18,19,20]. Concentrations ranged from 1 to 168 pg/mg and individual results are presented in Table 4.

Even though in these published cases, the doses and frequencies of consumption are unknown, it appears clearly that the subject did not use ostarine and cardarine on a limited number of occasions. His measured hair concentrations are much higher than those previously reported. This can be the reason why he experienced clinical troubles that improved after ostarine and cardarine discontinuation.

## 6. Conclusions

The recent widespread of new drugs all over the world via Internet has demonstrated that there is a need of analytical approaches to document these new habits of consumption. Therefore, implementing tests for SARMs seems of great importance, not only for anti-doping purposes, but also for clinical toxicologists or poison centres, in order to correctly establish a diagnosis of addiction or to document unusual side effects. Given the increasing popularity of SARMs abuse, vigilance and identification of new cases are required. As hair allows retrospective and long-term investigations, these specimens present all suitable properties to be qualified for SARMs or PPAR- δ detection, as the long-term abuse of these drugs can be harmful and potentially life-threatening [32]. Even though liver injury or rhabdomyolysis from SARMs and PPAR- δ have not been reported frequently, one can anticipate that this may be observed more often as the abuse of these drugs increases in the athletic and aesthetic markets.

## Figures and Tables

**Table 1:** Mass spectrometric and chromatographic data for the tested compounds.

Drug	Retention Time min	Transitions *m*/*z*	Collision Energy eV	Cone Voltage V
Ostarine	2.64	388.1 > 117.9 388.1 > 269.1	18 14	8 8
Cardarine	3.16	452.1 > 137.9 454.0 > 257.0 (+)	30 26	58 18
Bicalutamide-d_4_	2.57	433.1 > 255.1	14	14

**Table 2:** Validation parameters in blood and hair.

Parameters	Ostarine	Cardarine
Linearity in blood	1 to 1000 ng/mL	5 to 1000 ng/mL
Linearity in hair	0.5 to 1000 pg/mg	1 to 1000 pg/mg
Limit of detection in blood	0.2 ng/mL	1 ng/ml
Limit of detection in hair	0.1 pg/mg	0.3 pg/mg
Limit of quantitation in blood	1 ng/mL	5 ng/mL
Limit of quantitation in hair	0.5 pg/mg	1 pg/mg
Precision in blood (50 ng/mL)	12.8%	14.6%
Precision in hair (100 pg/mg)	11.9%	12.4%

**Table 3:** Measured concentrations in biological specimens from the admission.

Biological Specimens	Ostarine	Cardarine
Blood	1 ng/mL	403 ng/mL
Urine	88 ng/mL	Not detected
Hair	1105 pg/mg	146 pg/mg

**Table 4:** Ostarine concentrations in human hair reported in the literature.

Case	Purpose of the Test	Concentrations	References
1	Doping challenge	26 and 89 pg/mg in 2 × 3 cm	[9]
2	Doping challenge	6, 9, 4, 2, 2 and 1 pg/mg in 6 × 1 cm	[9]
3	Drug trafficking	3, 8, 14 and 21 pg/mg in 4 × 2 cm	[18]
4	Doping challenge	12 and 138 pg/mg, in 2 × 3 cm	[19]
5	Drug trafficking	146, 168, 93 and 101 pg/mg in 4 × 3 cm	[20]

## Data Availability

All data are available upon request sent directly to the corresponding author.
